# Splenic lymphangiomas as a common indication for splenectomy: a case series with literature review

**DOI:** 10.1186/s12893-022-01898-0

**Published:** 2022-12-29

**Authors:** Boubacar Efared, Aïchatou Balaraba Abani Bako, Hama Younssa, Idrissa Boubacar, Aliou Zabeirou, Hamadou Halidou Koura, Habiba Salifou Boureima, Soumaila Amadou, Idrissa Seriba Coulibaly, James Didier Lassey, Hassan Nouhou

**Affiliations:** 1 Faculté des Scientes de la Santé (FSS), Université Abdou Moumouni, BP: 10896, Niamey, Niger; 2 Laboratoire de cytologie et d’anatomie pathologiques, Hôpital National de Niamey, Niamey, Niger; 3 Hôpital Général de Référence, Niamey, Niger; 4 Service de chirurgie générale et viscérale, Hôpital National Amirou B. Diallo, Niamey, Niger; 5 Service de Chirurgie générale et viscérale, Hôpital National de Niamey, Niamey, Niger; 6 Hôpital Général de Référence, Maradi, Niger

**Keywords:** Spleen, Lymphangioma, Splenectomy, Cysts, Histopathology

## Abstract

**Background:**

Splenic lymphangiomas (SL) are very rare benign cystic lesions found in pediatric population. Their occurrence in adults is exceptional. Splenectomy is the common management of splenic lesions for diagnostic and/or therapeutic purpose. Our aim is to report additional cases of SL diagnosed on splenectomy specimens at our Pathology laboratory with literature review.

**Methods:**

This is a retrospective study including all cases of splenectomy recorded at our Pathology laboratory (June 2020–August 2022). We performed a comparison of clinicopathological features between patients with SL and those with other benign splenic diseases.

**Results:**

Sixteen cases of splenectomy were included. The mean age was 30.25 years (range of 6–70 years). The final histopathological diagnoses were congestive spleens in all cases of sickle cell disease (SCD) (5/16 patients, 31.25%), splenic cystic lymphangiomas (4/16 patients, 25%), capsular splenic infiltration by gastric and colic cancers (3/16 cases, 18.75%), splenic abscess (2/16 cases, 12.5%) and splenic rupture with subcapsular hematoma (1/16 patients, 6.25%). 12/16 patients (75%) had benign splenic conditions (4/12 with SL, 5/12 with SCD, 2/12 with abscess and 1/12 with splenic trauma). Patients with SL were older than those with other benign splenic conditions (mean age of 28.27 years versus 20.87 years). Also patients with SL presented with massive splenomegaly (mean splenic weight of 1675 g versus 418.75 g, mean splenic size of 19.62 cm versus 14.63 cm). Open surgery was performed in 15/16 patients (93.75%).

**Conclusion:**

Unlike previous studies, our series shows that SL are a common indication for splenectomy and occur in older patients with massive cystic splenomegaly. Open splenectomy is still an usual surgical practice in our country.

## Background

Splenic lymphangioma (SL) is a very rare cystic lesion affecting usually children and less commonly reported in adult persons [1–7]. These lesions are benign and considered by some authors as vascular (lymphatics) malformations rather than true tumors as they are found mainly in children and young patients with some congenital malformative syndrome such as Klippel-Trenaunay syndrome (association of varicose veins, cutaneous capillary malformations and hypertrophy of bone and/or soft tissue) [7–9]. SL may be asymptomatic or present with upper left abdominal pain, splenomegaly, hypersplenism or splenic rupture with hemorragic shock [1, 2, 10, 11]. Clinical and radiological features of SL are not specific, usually they present as cystic splenic lesions that may correspond to a variety of splenic diseases: congenital epithelial cysts (CEC), neoplastic cysts, parasitic hydatid cysts, traumatic cysts or splenic abscess [6, 7, 12–15].

Splenectomy either total or partial, laparoscopic or open, is the common management of splenic lesions for diagnostic and/or therapeutic purpose [4, 16–18], without the need for preoperative histopathological diagnosis through biopsy or fine-needle aspiration cytology (FNAC) [19]. Splenectomy is also a common therapeutic options in splenic trauma or in many hematologic diseases like sickle cell disease (SCD), thalassemia, hemolytic anemia, immune thrombocytopenia, hereditary spherocytosis or certain types of leukemias [18, 20, 21].

As the current literature offers only some case reports and rare case-series of SL, our aim is to report additional cases of SL diagnosed on splenectomy specimens at our newly operative pathology laboratory (the unique functional pathology laboratory in a public hospital in our country), with comparison of clinicopathological features between SL and other spleen diseases that have been the indications for splenectomy.

## Methods

### Study design

This is a retrospective study including all cases of splenectomy recorded at the Pathology laboratory of the Niamey National Hospital (Hôpital National de Niamey) from June 2020 to August 2022. As the unique Pathology laboratory in public hospital (functional since June 2020), we receive specimens from all medical centers of the country. Our cases of splenectomy were from 3 main National hospitals of the capital city of our country (Hôpital National de Niamey, Hôpital Général de Référence de Niamey, and Hôpital National Amirou Boubacar Diallo de Niamey) and 1 regional hospital (Centre Hospitalier Régional de Tillabéry). We have collected patients data such as: age, sex, preoperative diagnosis (indication for splenectomy), spleen weight and size (measured at our Pathology laboratory after surgical resection), splenic gross features, the type of surgical resection (either splenectomy alone or associated with other organs resection) and histopathological final diagnosis.

### Diagnoses

All cases have been diagnosed by routine histopathological techniques performed on surgical resected splenic specimens: formalin fixation, paraffin-embedding, microtome sectioning (4 microns thick) and hematoxylin–eosin (HE) staining. All cases have been analysed by using optic microscopy.

### Data analysis

Continuous variables are presented as means and ranges, categorical variables as percentages. We have grouped benign splenic diseases into 2 groups: a group of splenic lymphangioma (SL) and a group of ther benign splenic conditions. We performed a comparison between these 2 groups. In the comparison, we have excluded spleens resected for malignancies as they had all normal gross features, the indications were not primary spleen malignancies and the splenic parenchyma was not involved by the tumors (only superficial involvement of the capsule in some cases).

All descriptive statistical analyses were performed by using IBM SPSS Statistics 23.0.

## Results

### Overral clinicopathological features of the entire series

**Fig. 1:**
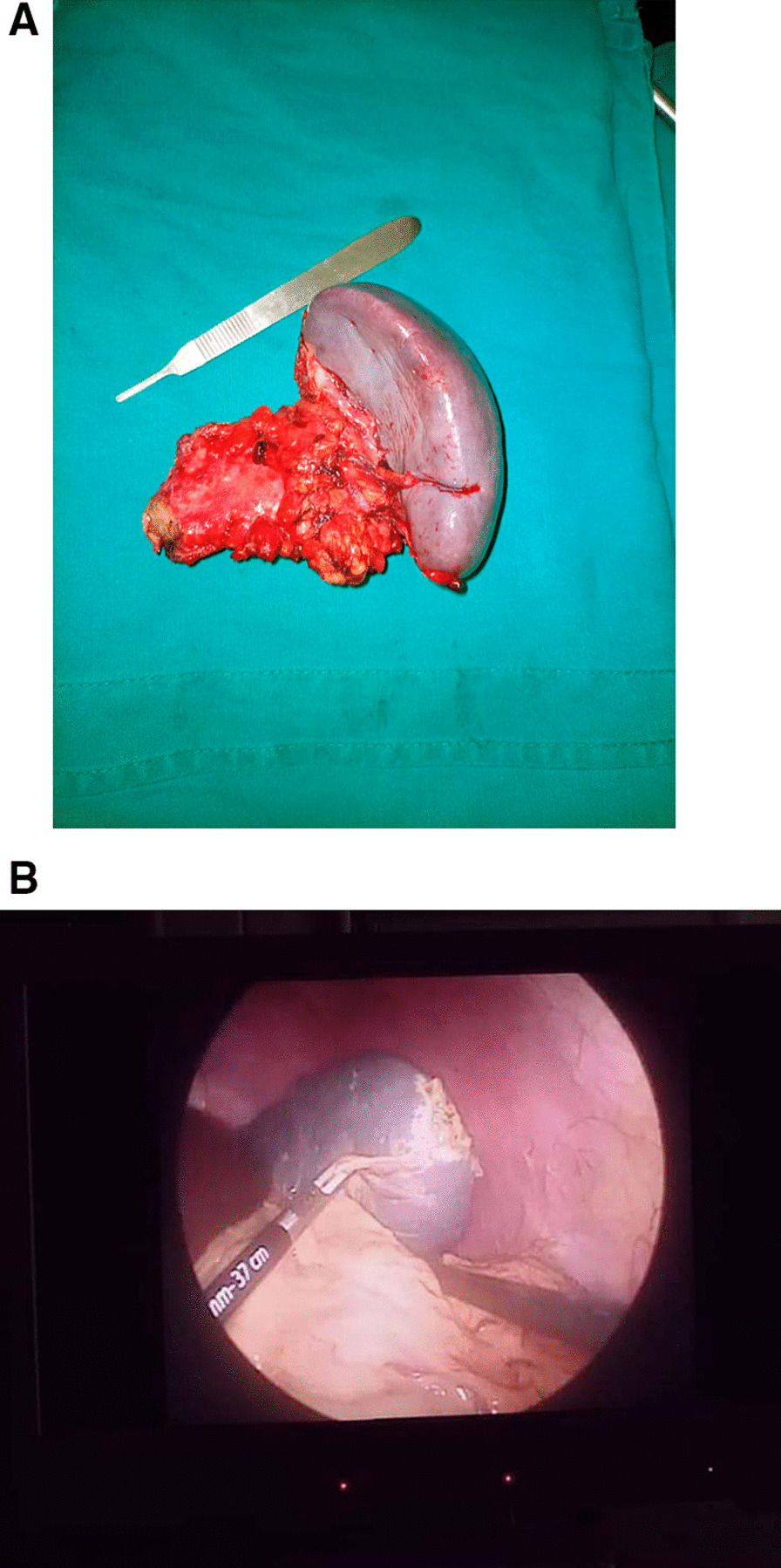
**A** Surgical specimen of distal splenopancreatectomy showing a spleen with a smooth surface (case 1). **B** Laparoscopic view of a spleen with a smooth surface in a patient with SCD (case 6)

**Fig. 2:**
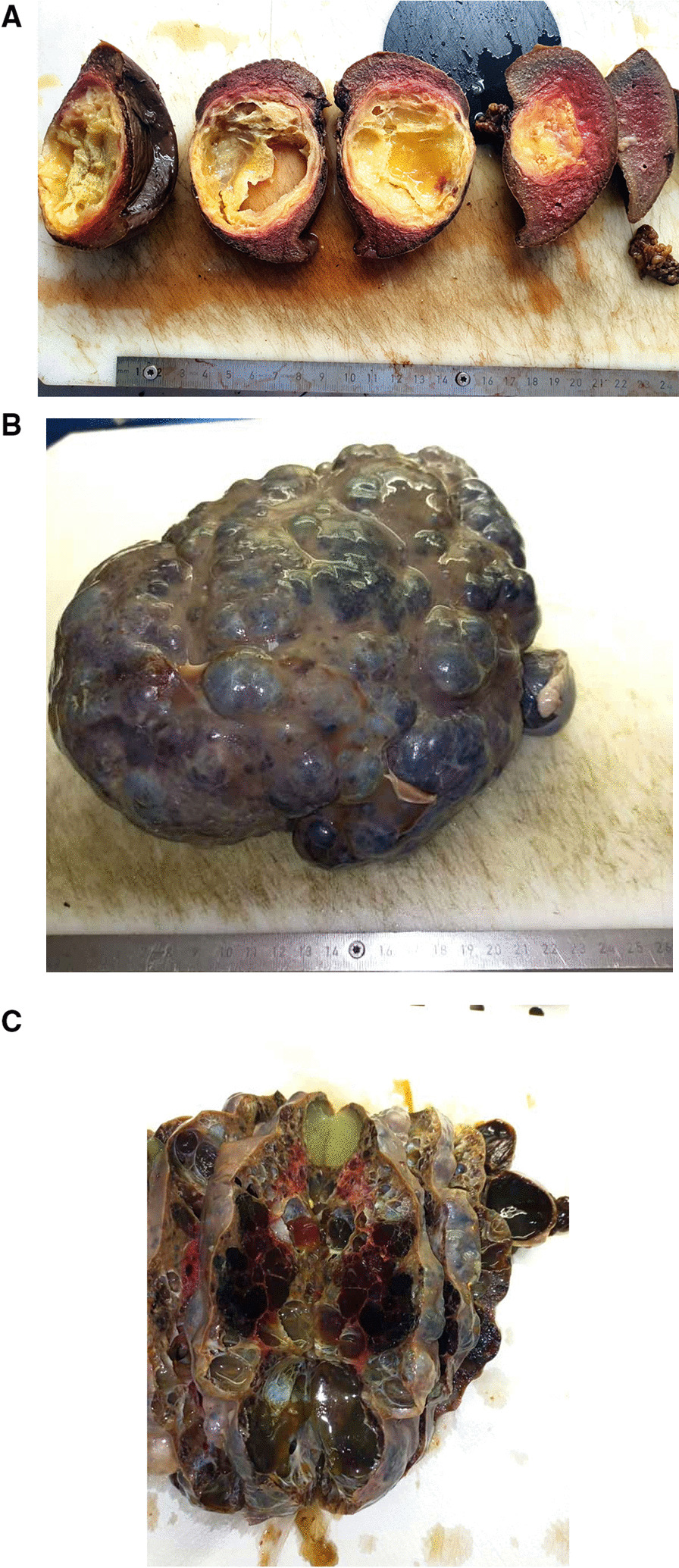
**A** Macroscopic view (after formalin fixation) of a splenic lymphangioma showing a well-encapsulated septated cyst with serous content (case 7). **B** Macroscopic view (after formalin fixation) of a splenic lymphangioma showing an entirely bosselated spleen (case 10). **C** Macroscopic view of a splenic lymphangioma cut surface showing multiple cysts occupying the entire spleen with mucoid and sero-hematic content (case 10)

The Table 1 summerises the clinicopathological features of our series. From June 2020 to August 2022, we have registered 16 cases of splenectomy. The mean age was 30.25 years (range of 6–70 years), with a male to female ratio (M:F) of 1,66 (10:6). The preoperative indications were hypersplenism and abdominal pain in patients with SCD (5/16 cases, 31.25%), splenic cystic lesions (4/16 cases, 25%), intra-abdominal cancers with suspected extension to the spleen (4/16 cases, 25%), 2 cases of isolated left upper abdominal pain with fever in 1 patient (2/16 cases, 12.5%) and 1 patient with abdominal trauma (1/16 cases, 6.25%). The mean splenic weight and size were respectively 837.5 grammes (g) (range of 100–2300 g) and 14.96 cm (range of 10–24.5 cm). Mainly, the gross features were homogenous congestive spleen with smooth surface in 9 cases (56.25%) (Fig. 1A, B) and multiloculated cystic lesions in 4 cases (25%) (Fig. 2A–C). All cases, except one (case 6 who underwent laparoscopic splenectomy and cholecystectomy) have been resected by open surgery (15/16 patients, 93.75%). Four cases (25%) underwent complex organs resections due to locally advanced tumors (Fig. 1A), in the remaining cases (12/16 cases, 75%) total splenectomy was performed.

**Table 1:** Clinicopathological features of our series (16 patients)

Cases	Age (year)	Sex	Preoperative diagnosis	Spleen weight (g)	Spleen size (cm)	Spleen gross features	Surgery	Histological diagnosis
1	33	F	Distal pancreatic tumor	–	12.5	Smooth surface, with normal parenchymal aspect	Distal splenopancreatectomy	Pancreatic neuroendocrine tumor, without spleen infiltration
2	55	F	Gastric tumor	–	11	Splenic surface involved by the gastric tumor	Partial gastrectomy + distal splenopancreatectomy	Gastric GIST involving the splenic capsule
3	64	M	Left colon tumor	–	10	Splenic surface involved by the colic tumor	Left colectomy + splenectomy	Colic adenocarcinoma involving the splenic capsule
4	35	M	Spleen abscess, hydatid cyst	2300	24	Unicystic lesion involving the entire spleen with yellowish compact content	Total splenectomy	Splenic lymphangioma
5	24	M	Splenic cyst	1300	17	Unicystic lesion involving the entire spleen with sero-hematic content	Total splenectomy	Splenic lymphangioma
6	15	M	Sickle cell disease (abdominal pain, gallstones)	200	11	Congestive spleen	Laparoscopic splenectomy + cholecystectomy	Congestive spleen + chronic cholecystitis
7	37	M	Splenic cyst	1500	13	Spleen centered by a 7 cm well-encapsulated septated cyst with serous content and mural calcifications	Total splenectomy	Splenic lymphangioma
8	52	M	Gastric carcinoma	-	12	Splenic surface involved by the gastric tumor	En-bloc gastrectomy with spleen, partial resection of pancreas, liver and colon	Gastric carcinoma with infiltration of the spleen capsule
9	17	M	Sickle cell disease (hypersplenism)	500	18	Congestive spleen	Total splenectomy	Congestive spleen
10	17	F	Splenic cystic lesion	1600	24.5	Multiple cysts with involvement of the entire spleen, mucoid and sero-hematic content	Total splenectomy	Splenic lymphangioma
11	14	F	Splenomegaly (fever, abdominal pain)	200	12	Ruptured cyst lined by fibrin deposits	Total splenectomy	Splenic abscess
12	12	M	Sickle cell disease (hypersplenism)	800	17	Congestive spleen	Total splenectomy	Congestive spleen
13	6	F	Sickle cell disease (hypersplenism)	200	11	Congestive spleen	Total splenectomy	Congestive spleen
14	19	M	Abdominal trauma	500	15.5	Splenic rupture with 5 cm supcapsular hematoma	Total splenectomy	Splenic rupture with subcapsular hematoma
15	70	M	Abdominal pain	100	10	Focal fibrous focis	Total splenectomy	Spleen abscess
16	14	F	Sickle cell disease (hypersplenism)	850	21	Congestive spleen	Total splenectomy	Congestive spleen

**Fig. 3:**
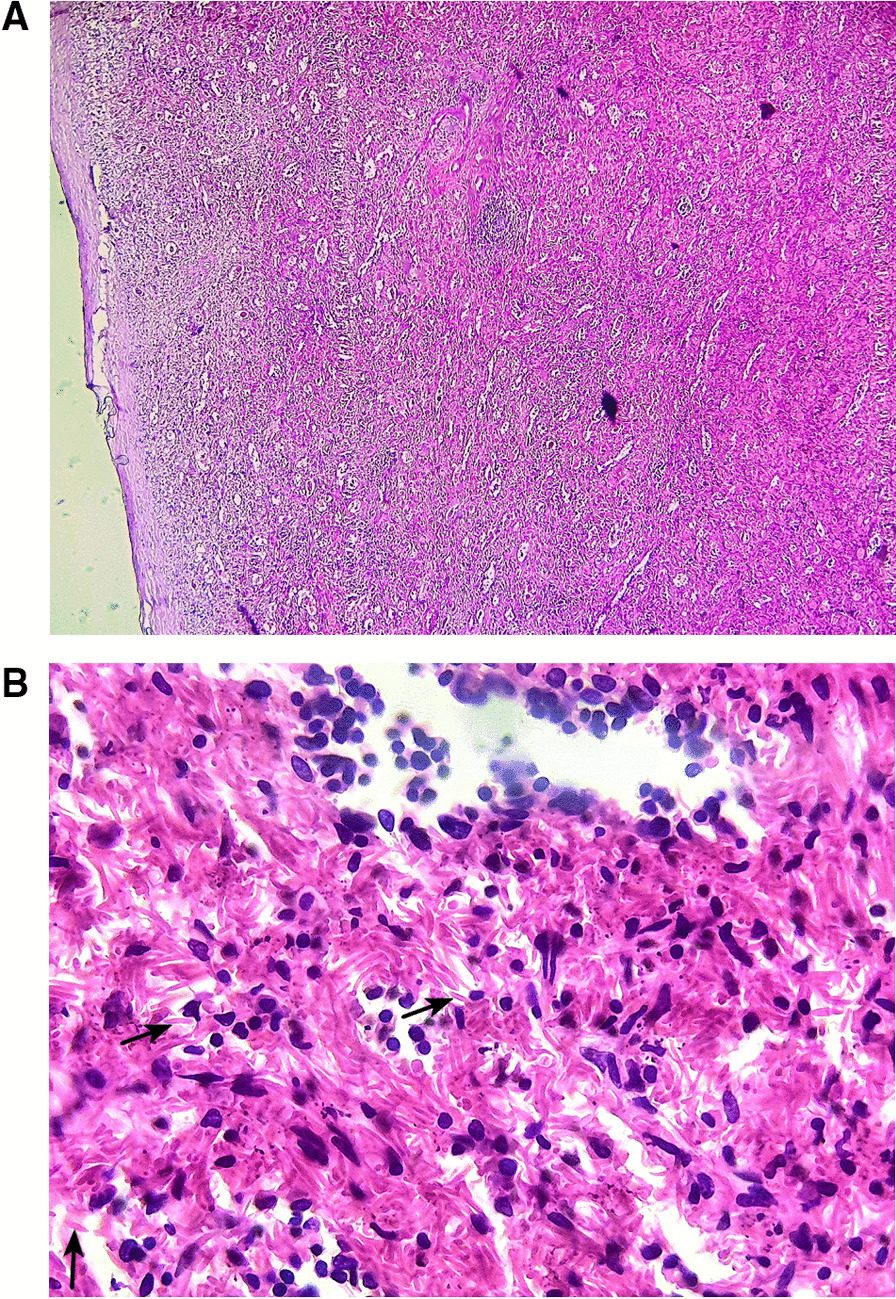
**A** Histological view of a splenic specimen in a patient with SCD showing congestive spleen with multiple dilated small vessels in the red and white pulp (hematoxylin–eosin × 40) (case 16). **B** At higher magnification, the splenic parenchyma is dissociated by numerous sickled, spindle-shaped red blood cells characteristic of the SCD (black arrows) (hematoxylin–eosin × 400) (case 16)

**Fig. 4:**
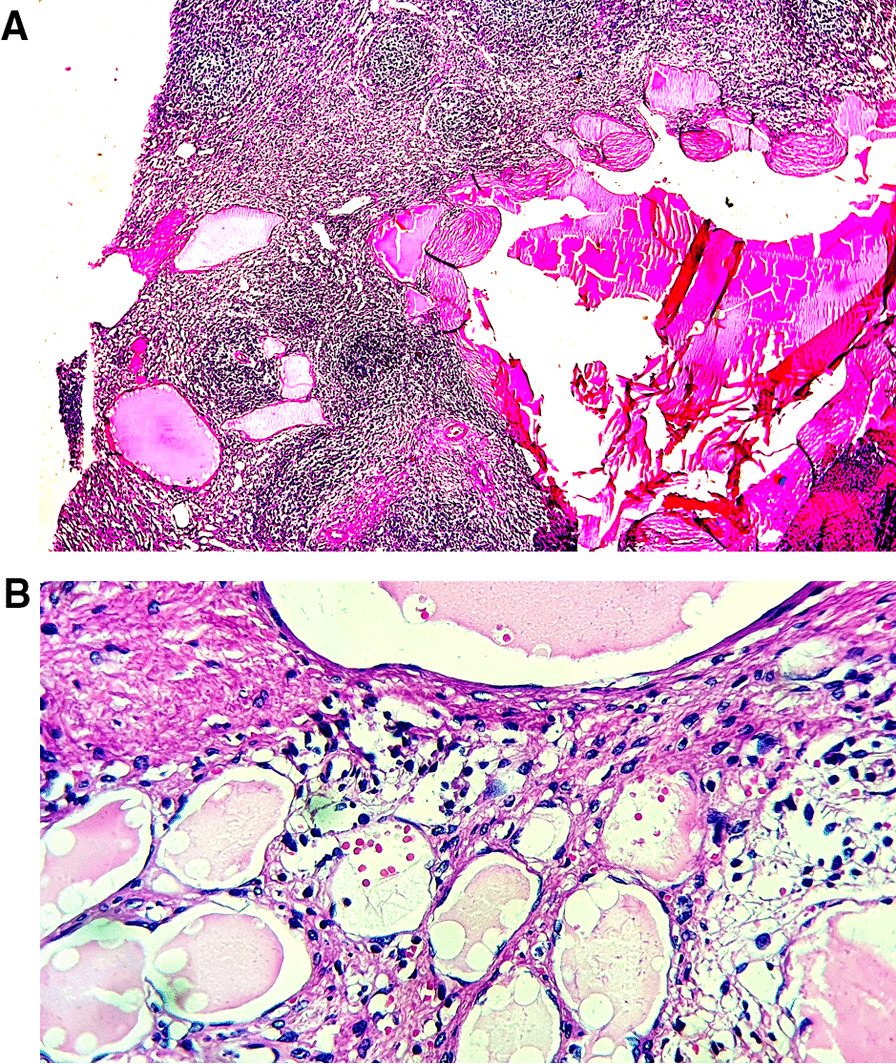
**A** Histological view of a splenic lymphangioma showing variable-sized cystic spaces containing amorphous eosinophilic material (hematoxylin–eosin × 100) (case 10). **B** At higher magnification, splenic lymphangioma is made of cystic spaces lined by flattened endothelial cells without atypias (hematoxylin–eosin × 400) (case 10)

**Fig. 5:**
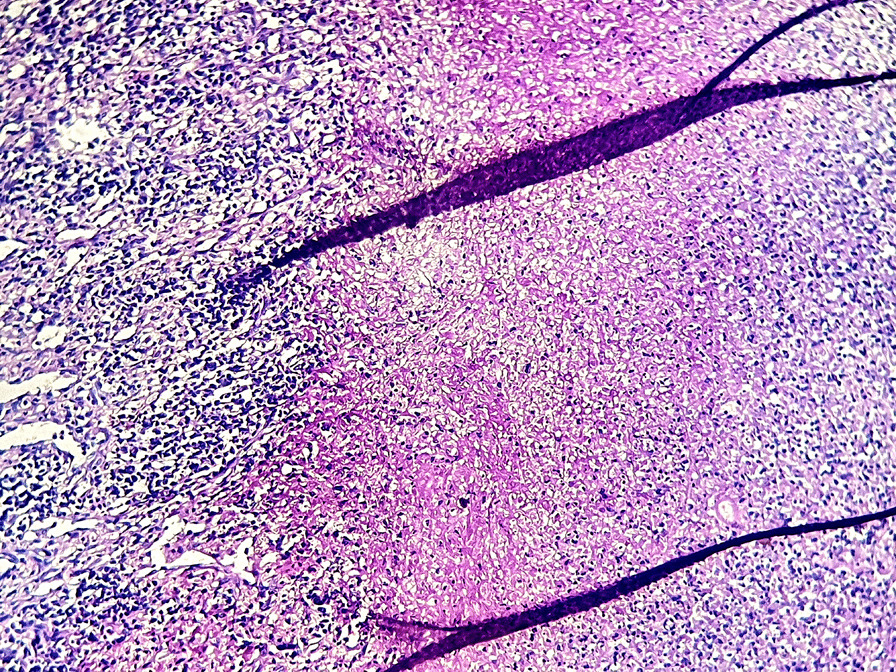
Histological image of a splenic abscess consisting of necrotic cells, altered neutrophils associated with fibrin deposition (hematoxylin–eosin × 200) (case 11)

The final histopathological diagnoses were congestive spleens in all cases of SCD (5/16 patients, 31.25%) (Fig. 3A, B), splenic cystic lymphangiomas in 4 patients (25%) (Fig. 4A, B), 3 cases of capsular splenic infiltration (18.75%) by a gastric GIST (gastrointestinal stromal tumor), colic adenocarcinoma and gastric adenocarcinoma, 2 cases of splenic abscesses (12.5%) (Fig. 5) and 1 patient with splenic rupture and subcapsular hematoma due to an abdominal trauma.

### Benign splenic diseases (conditions) versus splenic lymphangiomas

Our series included 4 cases (25%) of spleen resections associated with malignant tumors of adjacent organs (stomach, colon and pancreas) that superficially invaded the spleen capsule. The gross features of the spleen (weight and size particularly) in these cases were quite normal. The remaining cases (12/16 cases, 75%) had benign splenic conditions (4 patients with SL, 5 patients with SCD, 2 patients with abscesses and 1 case of splenic trauma).

The Table 2 shows the differential clinical and gross features between patients with SL and those with other benign conditions. Patients with SL were older than those with other benign splenic conditions (mean age of 28.27 years versus 20.87 years). Also cases of SL presented with massive splenomegaly with larger size and heavier spleens (mean splenic weight of 1675 g versus 418.75 g and mean splenic size of 19.62 cm versus 14.63 cm). There was a male predominance in patients with benign conditions, as well as in the 2 subgroups of SL and other benign diseases other than lymphangiomas.

**Table 2:** Comparison of clinicopathological features between patients with splenic lymphangiomas and those with other splenic benign conditions

	Overral benign conditions (n = 12)	Other diseases (n = 8)	Lymphangiomas (n = 4)
Mean age [range] (years)	23.33 [6–70]	20.87 [6–70]	28.25 [17–37]
Sex ratio (M:F)	8:4	5:3	3:1
Mean spleen weight [range] (g)	837.5 [100–2300]	418.75 [100–850]	1675 [1300–2300]
Mean spleen size [range] (cm)	16.16 [10–24.5]	14.43 [10–21]	19.62 [13–24.5]

## Discussion

We report from a Subsaharan African country a series of patients that underwent total splenectomy for a variety of diseases. Open surgery was performed in almost all cases (15/16 patients, 93.75%); SCD (5/16 cases, 31.25%), SL (4/16 cases, 25%) and malignant intra-abdominal tumors with suspected extension to the spleen (4 cases, 25%), were the most indications for the splenectomy. Benign splenic conditions were the most frequent indications for surgery (12/16 patients, 75%), among them SCD (5/12 cases, 41.66%) and SL (4/12 cases, 33.33%) were the leading causes of surgery. Hematologic diseases have been reported to be the major indication for splenectomy in the literature [17, 21–28], however we are not aware of SL as a common indication for surgery even in selected series (especially in pediatric population). The small sample and the short period of our study could be the reason of these discrepancies with the previous reports in the literature. Hematologic diseases such as idiopathic thrombocytopenia purpura (ITP) and hereditary spherocytosis are the major indication for splenectomy in series especially from Western countries [21–23, 25] while splenic complications of SCD represent a common cause of splenectomy in African and Middle-East countries [16, 29, 30]. Splenectomy is usually performed in patients with SCD complications like acute splenic sequestration crisis, hypersplenism, splenic abscess, and massive splenic infarction, with good outcomes and this surgery also reduces requirements for repeated blood transfusions and prevents mechanical complications of an enlarged spleen [16, 30]. However, a recent meta-analysis challenged these beneficial effects of splenectomy by concluding that there is a lack of evidence from trials showing that splenectomy improves survival and decreases morbidity in people with SCD [31]. Malignant tumors either primary or secondary affect very rarely the spleen, and represent a less usual indication for splenectomy [23, 32]. In our series there were no primary splenic tumors, however we have registered 3 cases with splenic capsular invasion by cancers from adjacent organs (stomach and left colon).

Since its introduction in surgical clinical practice in early 1990 s, laparoscopic splenectomy (LS) has become a gold standard in the management of patients with splenic diseases either benign or malignant [21, 23, 25]. Many reports shows that LS has lower morbidity with cosmetic advantages, less perioperative bleeding, short hospital stay and less conversion rate to open surgery [23, 33]. Even in patients with massive splenomegaly (> 1000 g), LS could be safely performed [33, 34]. However, this minimally invasive surgery requires experience and acquisition of more technical skills over time (learning curve), with longer operative time especially in patients with massive splenomegaly [23, 25, 35]. In our series, open splenectomy (OS) was widely performed (15/16 cases, 93.75%) even in patients with benign diseases and mild splenomegaly (< 500 g). The reason for this classic surgical approach was the lack of adequate laparoscopic materials in our hospitals, with rare surgeons that have skills in laparoscopic surgery.

In our study, patients with SL were older than patients with other benign splenic conditions (mean age of 28.25 versus 20.87 years), because of many children with SCD in this subgroup of benign splenic conditions. Also, patients with SL presented with heavier spleens and massive splenomegaly (mean weight and size of 1675 g and 19.62 cm) whereas patients with other benigh splenic conditions had mild splenomegaly (mean weight and size of 418.75 g and 14.43 cm). In fact in this subgroup, the indications for splenectomy were acute symptoms such as abdominal pain, fever and hypersplenism, rather than the importance of the splenomegaly and abdominal distension in comparison with patients that had SL.

In the literature, SL are usually reported in children as asymptomatic or rarely symptomatic lesions, with few case reports in adults [2, 4, 6, 7, 15, 36]. Our current series challenged these classic features as all patients presented with massive splenomegaly with abdominal distension (mean splenic weight of 1675 g, range of 1300–2300 g). Also, in our series patients were older (mean age of 28.25 years, range of 17–37 years) with 3 young adults and 1 adolescent patient of 17 years. As in our cases, SL present as cystic lesions of variable size with honeycombing appearance and compact pale, yellowish content (lymphatic fluid) on resected specimens [2, 7]. The preoperative imaging techniques are not specific, they show only the cystic aspect of the lesion, that could correspond to a variety of cystic splenic lesions [12]. The Table 3 summerises the main characteristics of splenic cystic lesions. Several classifications of splenic cystic lesions have been proposed [6, 13, 15, 36]. Splenic cystic lesions are classified as: neoplastic cysts, congenital epithelial cysts (CEC), parasitic cysts (mainly hydatid cysts), and other cysts as a results of infarction (traumatic cysts) or inflammation/infection (abscess). Neoplastic cysts include vascular lesions (hemangiomas, lymphangiomas or angiosarcomas) or any malignant solid splenic tumors with cystic degeneration. Vascular cystic lesions are lined by regular flat endothelial cells, with luminal content made of red blood cells (hemangioma) or proteinaceous amorphous eosinophilic material (lymphangioma) [36]. The histological aspects of our 4 cases of SL were typically consistent with the later aspect. In angiosarcomas, the lining endothelial cells present atypias, mitoses and necrosis [37]. Congenital epithelial cysts (CEC) are common in pediatric population, the cysts are lined by epithelial cells with mesothelial, transitional or squamous differenciation. All these types of epithelial cells could be found in combination in a single lesion. These lesions are supposed to derive from mesothelial invagination through the splenic parenchyma [6, 15]. Splenic hydatid cysts are caused by *Echinococcus granulosus* infection and are endemic in certain areas of the World [38]. On histopathological analysis, the parasit’s structures are readily observed in the splenic cyst. The remaining causes of splenic cysts are the result of previous trauma, infarction or infection. They present histologically as pseudo-cystic lesions without epithelial or endothelial lining. Previously, splenic pseudo-cysts were believed to be common, however a careful histological analysis usually find focal endotheilal or epithelial lining in what may be misdiagnosed as a pseudo-cyst [14].

**Table 3:** Main causes of splenic cysts and their characteristic histopathological features

	Cystic wall/lining	Cystic content
Neoplastic cysts		
- Lymphangioma - Hemangioma - Cystified solid tumor	- Endothelial cells - Endothelial cells - Atypical tumor cells	- Compact lymphatic fluid - Red blood cells - Necrotic tumor cells
Congenital epithelial cysts		
- Mesothelial cyst - Epidermoid cyst - Transitional cyst	- Mesothelium - Squamous cells - Transitional cells	Liquid content with variable aspects
Parasitic cysts (Echinococcus)	Fibrous wall with reactive inflammatory cells	Parasitic structures
Other cysts (trauma, inflammation)		
- Pseudo-cyst - Abscess	- Fibrous wall with inflammatory cells - Fibrous wall and inflammatory cells	- Variable necrotic and inflammatory cells - Altered neutrophils, necrotic cells, fibrin deposition

As a retrospective analysis, our study has some limitations, especially a small number of patients. The data about imaging techniques and follow-up are not available. However some provisional conclusions could be drawn, until the availability of larger studies on SL in the future.

## Conclusion

Unlike previous literature reports, our series shows that splenic lymphangiomas (SL) are a common indication for splenectomy. They occur in older patients and present with massive cystic splenomegaly. Open splenectomy is still an usual surgical practice in poor countries. The definitive diagnosis of SL relies on the histopathological analysis of the resected splenic specimens.

## Data Availability

All data generated or analysed during this study are included in this article.

## Abbreviations

SL: Splenic lymphangioma
SCD: Sickle cell disease
HE: Hematoxylin–eosin
FNAC: Fine needle aspiration cytology
GIST: Gastrointestinal stromal tumor
LS: Laparoscopic splenectomy
ITP: Idiopathic thrombocytopenia purpura
CEC: Congenital epithelial cysts
